# Spotting the difference: short versus long RP tachycardia explained

**DOI:** 10.1016/j.hrcr.2024.09.004

**Published:** 2024-10-15

**Authors:** Margaret Harvey

**Affiliations:** University of Tennessee Health Science Center, College of Nursing; Department of Acute and Tertiary Care, Memphis, Tennessee

**Keywords:** Supraventricular tachycardia, Atrioventricular node reentry tachycardia, Short RP tachycardia, Long RP tachycardia, Typical AVNRT, Atypical AVNRT

## Introduction

Atrioventricular nodal reentrant tachycardia (AVRNT) is a form of supraventricular tachycardia (SVT) classified as either “typical” or “atypical.” Most cases are typical with a short RP interval, so it is important to recognize associated electrocardiographic (ECG) features, conduction patterns, and how it is distinguished from atypical AVNRT with a long RP interval.^1^ This case report describes a patient with ECG findings consistent with short RP tachycardia and electroanatomic mapping of typical AVNRT.

## Case Report

**Figure 1:**
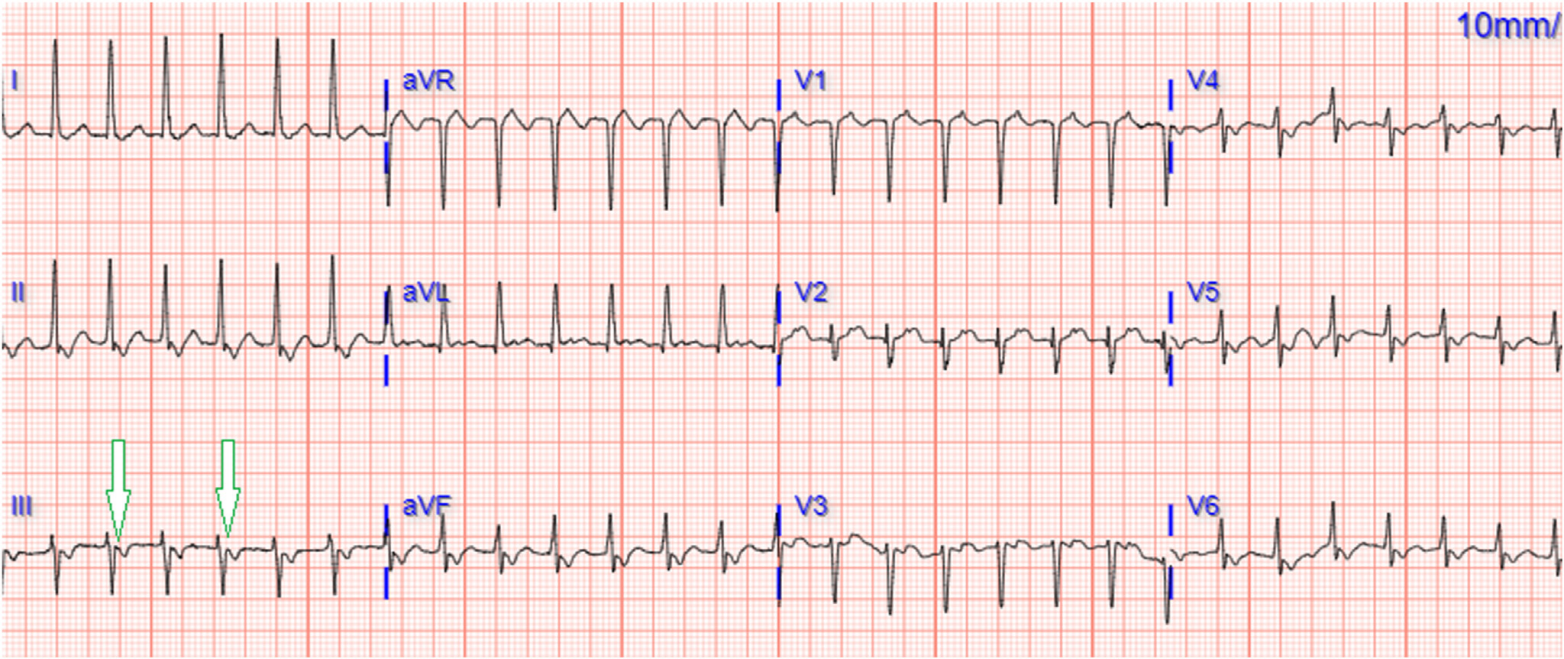
Twelve-lead electrocardiogram with long RP tachycardia and 1:1 conduction. Note the retrograde P waves at the end of the QRS complex in the inferior leads.

A 52-year-old female patient with a history of palpitations secondary to documented paroxysmal SVT was admitted for palpitations and shortness of breath. The initial 12-lead ECG showed a regular, narrow complex, short RP tachycardia at a rate of 169 beats per minute and 1:1 conduction (Figure 1). Adenosine 6 mg was administered with termination of the SVT and restoration of normal sinus rhythm, with no evidence of preexcitation. The patient underwent an electrophysiology study and mapping with radiofrequency ablation (RFA) of the slow atrioventricular nodal pathway for inducible typical AVNRT.

## Discussion

For SVTs that are AV node-dependent with a reentrant circuit such as AVNRT, the RP interval can be useful in distinguishing the type of arrhythmia. In typical AVNRT, conduction follows the slow-fast pathway, with atrial and ventricular activation occurring almost simultaneously.^1^ As a result, the P wave is hidden in the QRS complex or may be seen at the end of the QRS complex as a negative deflection in the inferior leads and as a slightly positive R’ in lead V1.^2^ This is referred to as a “short RP” tachycardia because the P wave is closer to the prior QRS complex than the subsequent QRS complex.^2^ Atrioventricular reentry tachycardia (AVRT) may also present as a “short RP” tachycardia, as seen in orthodromic AVRT where the reentrant circuit follows an antegrade conduction through the AV node with subsequent retrograde conduction over an accessory pathway. This produces a retrograde or negative P wave that can be seen at the beginning of the ST segment and is closer to the prior QRS complex than the subsequent QRS complex.^2^

In atypical AVNRT, the conduction may follow a fast-slow pathway and the P wave is closer to the subsequent QRS complex. This is called a *long RP tachycardia* because the P wave is farther from the prior QRS complex and closer to the subsequent QRS complex. Long RP tachycardia can also be seen in an unusual form of AVRT called permanent junctional reciprocating tachycardia (PJRT). This form of SVT involves an unusual accessory bypass tract with retrograde conduction, resulting in delayed atrial activation and lengthening the RP interval such that the P wave is closer to the subsequent QRS complex.^2^ Atrial tachycardia is also considered a *long RP tachycardia*, as the P wave originates in the atrium and follows the normal conduction pathway through the ventricle with the P wave preceding each QRS complex.

## Conclusion

In the presenting case, the patient had ECG findings consistent with the most common form of AVNRT with a short RP interval. Typical AVNRT was confirmed during the EP study, in which mapping showed conduction along the slow-fast pathway with subsequent successful RFA and termination of the arrhythmia. This case report describes how measurement of the RP interval can aid in differential diagnosis for typical AVNRT and AVRT as well as atypical AVNRT and AVRT. However, it should be emphasized that the specific conduction pattern and final diagnosis can be identified only through an electrophysiologic study and mapping.

## Disclosure

The author has no conflicts of interest to disclose.
